# Terrestrial isopods of the family Eubelidae Budde-Lund, 1899 from Iran, with description of a new species (Isopoda, Oniscidea)

**DOI:** 10.3897/zookeys.801.23340

**Published:** 2018-12-03

**Authors:** Ghasem M. Kashani, Ahdiyeh Abedini, Giuseppe Montesanto

**Affiliations:** 1 Department of Biology, Faculty of Sciences, University of Zanjan, Zanjan, Iran University of Zanjan Zanjan Iran; 2 University of Pisa, Department of Biology, via A. Volta 4bis, 56126 Pisa, Italy University of Pisa Pisa Italy

**Keywords:** Eubelidae, Iran, new species, new records, Oniscidea

## Abstract

In the present work, terrestrial isopods of the family Eubelidae are investigated in Iran. The genera *Periscyphis* and *Somalodillo* are reported for the first time. More localities are presented for *Koweitoniscustamei* (Omer-Cooper, 1923) while *K.shafieii* Kashani, **sp. n.** is described and figured. A map indicating the sampling localities for the species is presented.

## Introduction

The family Eubelidae Budde-Lund, 1899 includes 50 genera distributed mostly in the tropical areas of Africa, and partly in south-eastern Asia and in the Arabian Peninsula (Taiti et al. 1991). Schmidt (2003) considered the occurrence of some eubelid species in the Neotropics due to human activities. Uropods with flattened sympodites and reduced exopodites inserting medially on the distal margin of the sympodites, presence of *sulcus arcuatus* on the lateral margins of the first coxal plates along with the conglobation ability are the most significant diagnostic characters of the family (Taiti et al. 1991; Schmidt 2003). *Koweitoniscustamei* (Omer-Cooper, 1923) was the first eubelid species reported from Iran (Kashani 2014). In the present study, more localities are reported for the species and two more species are identified: *Periscyphisvittatus* Omer-Cooper, 1926 and *Koweitoniscusshafieii* Kashani, sp. n. The genus *Somalodillo* is also reported for the first time based on one female specimen. Sampling localities are presented on a map.

## Material and methods

**Figure 1.**
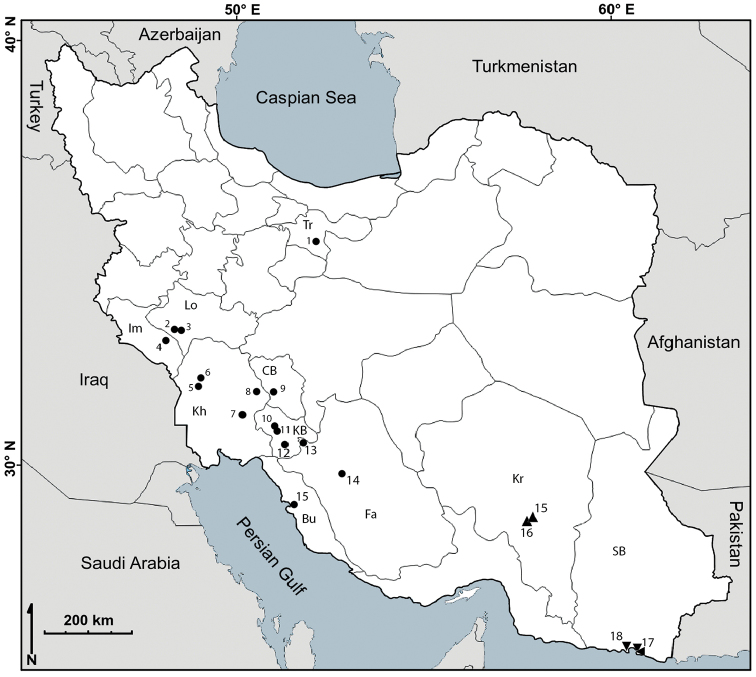
Map of Iran indicating the sampling localities of *Periscyphisvittatus* (▼), *Koweitoniscustamei* (●), *K.shafieii* (▲), and *Somalodillo* sp. (◄). Numbers refer to the sampling localities indicated in brackets in the material examined section for each species. Abbreviations: Bu: Bushehr; CB: Chehar–Mahal va Bakhtiari; Fa: Fars; Im: Ilam; KB: Kohgiluyeh va Boyer–Ahmad; Kh: Khouzestan; Kr: Kerman; Lo: Lorestan; SB: Sistan va Balouchistan; Tr: Tehran.

The material examined in the present study was collected in Iran since 2008 (Figure 1). The specimens were collected by hand and preserved in 96% ethanol. The specimens were dissected and body parts were slide-mounted using Euparal (Carl Roth, Karlsruhe). Drawings were made using a drawing tube on a Nikon Y-IDT compound microscope. Color images were taken using an Olympus DP71 digital camera on an Olympus SZH10 stereomicroscope. Type material of the newly described species is deposited in the Zoological Museum, University of Tehran (**ZUTC**), the Iranian Research Institute of Plant Protection (IRIPP) and in the personal collection of the first author (**PCGMK**). Drawing and plates were arranged with the methods described in Montesanto (2015, 2016).

## Systematic account

### Order Isopoda Latreille, 1817

#### Suborder Oniscidea Latreille, 1802

##### Family Eubelidae Budde-Lund, 1899

###### Genus *Periscyphis* Gerstaecker, 1873

**Type species.***Periscyphistrivialis* Gerstaecker, 1873 by monotypy.

####### Periscyphis vittatus

Taxon classificationAnimaliaIsopodaEubelidae

Omer-Cooper, 1926

Figure 2A

######## Material examined.

Sistan va Balouchistan, [17] 6 ♀♀, Chabahar, 4 Dec. 2008, leg. H. Salehi (PCGMK 1385); [18] 3 ♂♂, 10 ♀♀, 15 Km to Konarak, 25°26.3'N, 60°29.5'E, 9 Feb. 2009, leg. E. Entezari (PCGMK 1714); [18] 1 ♂, 1 ♀, 15 Km E Konarak, 25°26.3'N, 60°29.5'E, 9 Feb. 2009, leg. E. Entezari (IRIPP Iso.1066).

######## Remarks.

**Figure 2.**
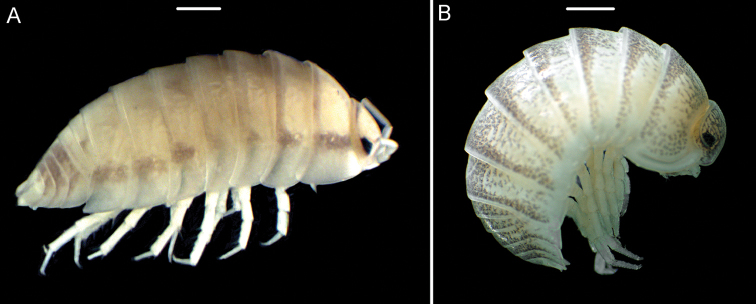
**A***Periscyphisvittatus***B***Koweitoniscustamei*. Scale bar: 1 mm.

The genus *Periscyphis* comprises 46 species, mostly present in eastern Africa and the Arabian Peninsula (Schmalfuss 2003; Taiti and Checcucci 2011; Taiti and Schotte 2016; Taiti and Montesanto 2018). *Periscyphisvittatus* is reported here for the first time from two localities in southern Iran (Figure 1). As reported for Pakistan (Schmalfuss 2003), this species is most probably introduced to Iran. The identification of the species was based on the comparison of the characters of the specimens (Figure 2A) with the description and illustration presented by Ferrara & Taiti (1986: 96; fig. 6).

######## Distribution.

Eritrea; Djibouti; Mozambique; Somalia; Arabian Peninsula; Socotra Island; Pakistan; Iran.

###### Genus *Koweitoniscus* Vandel, 1975

**Type species.***Koweitoniscusahmadii* Vandel, 1975 (= *Periscyphistamei* Omer-Cooper, 1923) by original designation and monotypy.

####### Koweitoniscus tamei

Taxon classificationAnimaliaIsopodaEubelidae

(Omer-Cooper, 1923)

Figure 2B

######## Material examined.

Boushehr, [15] 1 ♂, 1 ♀, Borazjan to Boushehr, 20 Km to Boushehr, 29°02.7'N, 51°03.0'E, 16 May 2008, leg. G.M. Kashani, (PCGMK 1224); Kohgiluyeh-va-Boyerahmad, [10] 1 ♂, Sough, 2 May 2003, leg. M. Rezaei (PCGMK 1271); 1 ♀, Sough, 2 May 2003, leg. M. Rezaei (IRIPP Iso-1006); [11] 1 ♀, Dehdasht, 2 May 2003, leg. M. Rezaei (PCGMK 1272); [12] 1 ♀, Gachsaran, Tappe-Aqrab, 3 May 2003, leg. M. Rezaei (PCGMK 1268); Cheharmahal-va-Bakhtiari, [9] 1 ♂, Sarkhoun to Lordegan, 31°40.3'N, 50°44.4'E, 18 June 2016, leg. G.M. Kashani, A. Abedini & Z. Hatami (PCGMK 2328); Lorestan, [2] 1 ♂, 1 ♀, Poldokhtar, 33°07.0'N, 47°43.4'E, 12 Nov. 2008, leg. G.M. Kashani (IRIPP Iso-1007); [3] 15 ♂♂, 20 ♀♀, Poldokhtar to Andimeshk, 80 Km to Andimeshk, 32°56.2'N, 47°52.5'E, 12 Nov. 2008, leg. G.M. Kashani (PCGMK 1395); [3] 1 ♂, 7 ♀♀, Poldokhtar to Andimeshk, 80 Km to Andimeshk, 32°56.2'N, 47°52.5'E, 12 Nov. 2008, leg. G.M. Kashani (IRIPP Iso-1008); Ilam, [4] 1 ♂, 1 ♀, Dehloran to Abdanan, 32°39.9'N, 47°32.0'E, 13 Nove. 2008, leg. G.M. Kashani (PCGMK 1399); Tehran, [1] 1 ♂, Varamin, Pishva, 35°12.4'N, 51°48.4'E, 26 Apr. 2009, leg. G.M. Kashani (PCGMK 1436); Khouzestan, [5] 5 ♂♂, 1 ♀, Shoush, Karkhe national park, 5 Aug. 2017, leg. H. Maddahi (PCGMK 2642); [6] 2 ♀♀, Shoush to Andimeshk, Safar Abad village, by the Dez river, 32°16.1'N, 48°24.0'E, 6 May 2009, leg. H. Salehi (IRIPP Iso-1010); [7] 9 ♂♂, 9 ♀♀, Ramhormoz, Bony village, 23 Mar. 2015, leg. M. Larti (PCGMK 2078); [8] 3 ♂♂, 9 ♀♀, Dehdez to Izeh, 22 Km to Izeh, 31°49.0'N, 50°03.0'E, 19 Jun. 2016, leg. G.M. Kashani, A. Abedini & Z. Hatami (PCGMK 2350); Fars, [13] 1 ♀, Kopen to Masiri, 2 Km to Dozak village, 30°18.5'N, 51°22.7'E, 19 Jul. 2015, leg. G.M. Kashani, Z. Hatami & A. Abedini (PCGMK 2207); [14] 1 ♂, 1 ♀, Shiraz, by the Parishan lake, 31 Oct. 2012, leg. S. Hosseini (IRIPP Iso-1011).

######## Remarks.

The broad distribution of *Koweitoniscustamei* in south and south-western Iran was reported by Kashani (2014). Here, more localities are introduced for the species (Figure 1). The presence of the species in the town of Tehran might be due to human activities. The identification of the species was based on the comparison of the species (Figure 2B) with the original descriptions and illustrations presented by Omer-Cooper (1923: 96, figs 1–16) and the figures by Ferrara & Taiti (1986: 100, fig. 15).

######## Distribution.

Syria; Iraq; Kuwait; Iran.

####### Koweitoniscus shafieii

Taxon classificationAnimaliaIsopodaEubelidae

Kashani sp. n.

http://zoobank.org/DC0328B2-6868-4361-B85D-D7FF7C49E55E

Figs 3 , 4 , 5

######## Material examined.

**Holotype**: ♂, 10 mm, [15] Kerman, Jebalbarez to Jiroft, 10 Km to Jiroft, 28°45.8'N, 57°45.5'E, elev. 835m, 25 Feb. 2009, leg. G.M. Kashani (ZUTC 6747). **Paratypes**: 1 ♀, same data as holotype (ZUTC 6748); 1 ♀, same data as holotype (IRIPP Iso.1009); 2 ♂♂, 4 ♀♀, same data as holotype (PCGMK 1450); [16] 1 ♂, Jiroft, 6 Apr. 2009, leg. E. Entezari (PCGMK 1440).

######## Diagnosis.

Head with an interrupted frontal ridge; inner lobe of schisma longer than outer one; telson with pointed distal part; male pleopod 1 endopodite bent inward with pointed apex.

######## Description.

Maximum length: male 10 mm; female 11 mm.

**Figure 3.**
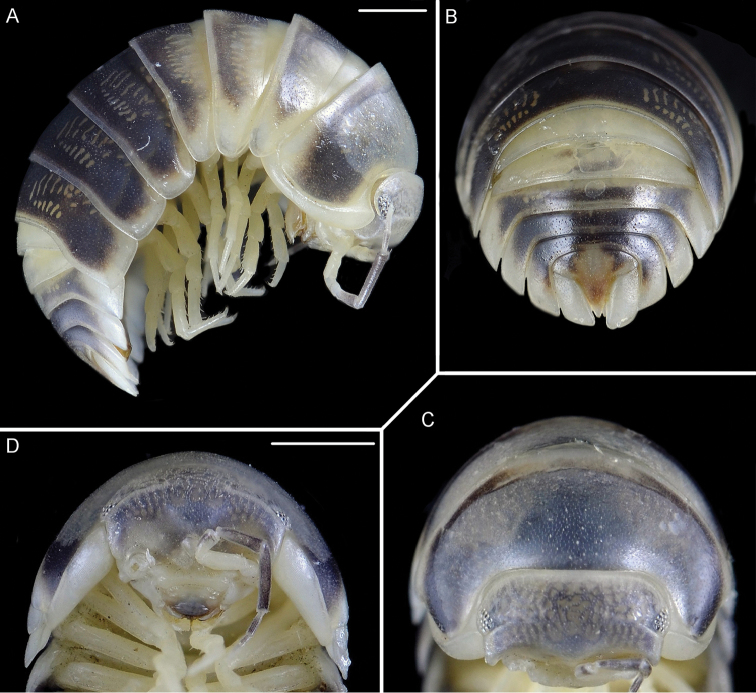
*Koweitoniscusshafieii* sp. n., female, paratype. **A** lateral view **B** pleon in dorsal view **C** cephalothorax and pereonite 1 in dorsal view **D** cephalothorax in frontal view. Scale bar: 1 mm.

**Figure 4.**
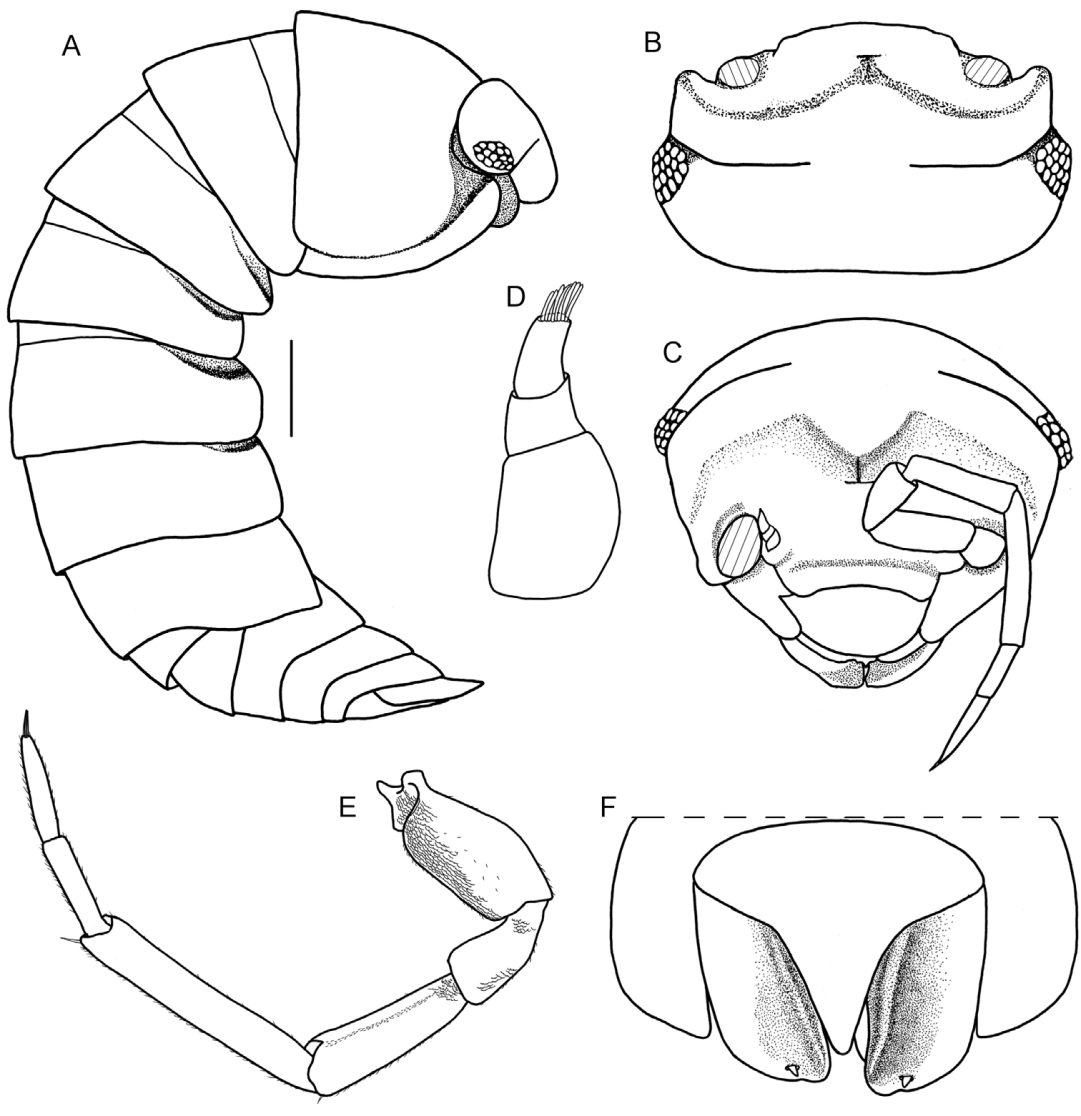
*Koweitoniscusshafieii* sp. n., paratype, female. **A** lateral view **B** cephalothorax in dorsal view **C** cephalothorax in frontal view **D** antennula **E** antenna **F** telson and uropods. Scale bar: 1 mm.

*Coloration*: vertex, pereon-tergites, pleon-tergites 3–5 and telson blackish; posterior margins of pereon-tergites II-III, pereon and pleon epimera, uropods, pleonites I-II and pereopods yellowish (Figure 3A). *Body* smooth, convex, able to roll up into a ball (Figs 3A, 4A).

*Cephalothorax* (Figs 3C, D; 4B, C) with frontal ridge broadly open in the middle; eyes with 16–18 ommatidia. Antennula (Figure 4D) with second article shortest; third article bearing a tuft of aesthetascs at apex. Antenna (Figure 4E) with fifth article of peduncle longer than flagellum; flagellum with two articles, proximal article as long as the distal one.

*Pereonite 1* (Figs 3A, 4A) with a wide *sulcus arcuatus* along the lateral margin; posterolateral corner with a schisma, inner and outer lobes rounded, inner lobe more protruding backwards than outer one; posterior margin straight.

*Telson* (Figs 3B, 4F) approximately 1.3 times as wide as long, with concave sides and distal part triangular with pointed apex. Uropod (Figs 3B, 4F) with subquadrangular protopodite, posterior margin slightly sinuous but not indented; exopodite minute inserted dorsally near the posterior margin.

**Figure 5.**
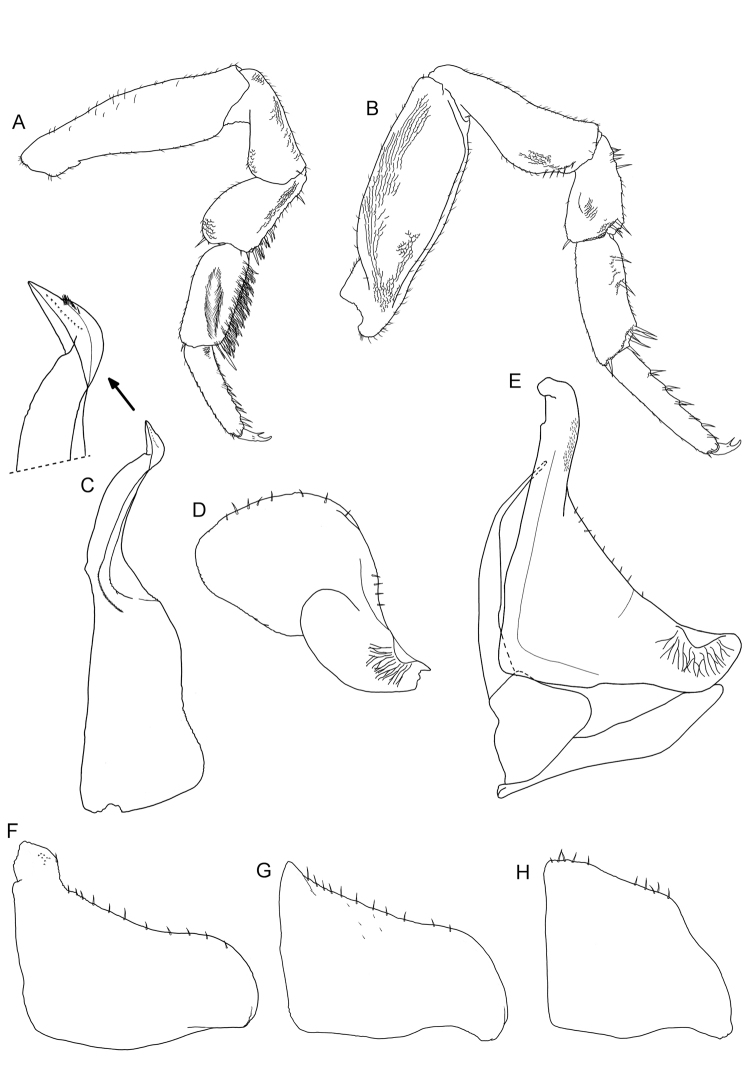
*Koweitoniscusshafieii* sp. n., male, paratype. **A** pereopod 1 **B** pereopod 7 **C** pleopod 1 endopodite with enlarged apex **D** pleopod 1 exopodite **E** pleopod 2 **F** pleopod 3 exopodite **G** pleopod 4 exopodite **H** pleopod 5 exopodite.

Male: Pereopods 1–4 carpus with a brush of pointed setae (Figure 5A). Pereopod 7 (Figure 5B) ischium narrow with concave ventral margin; merus and carpus elongated, without distinct specializations. Pleopod 1endopodite (Figure 5C) with medial part bent outward and distal part bent inward with pointed apex bearing a row of fine setae on outer margin; exopodite (Figure 5D) short, with widely rounded hind lobe equipped with a row of pointed setae. Pleopod 2 (Figure 5E) exopodite longer than wide with numerous small scales and a line of setae on the outer margin. Pleopod 3–5 exopodites as in Figs 5F–H.

######## Etymology.

The species is named after Dr. Soheila Shafiei, Ph.D. classmate of GMK, now a herpetologist in Shahid Bahonar University of Kerman, Iran.

######## Remarks.

The genus *Koweitoniscus* presently embraces five species (Schmalfuss 2003; Taiti and Checcucci 2011; Kashani 2014, Taiti and Montesanto 2018): *K.tamei* (Omer-Cooper, 1923) from Syria, Iraq, Kuwait and Iran, *K.rostratus* Ferrara & Taiti, 1986 from south-western Saudi Arabia, *K.vanharteni* Ferrara & Taiti, 1996 from Yemen, *K.korshunovi* Taiti & Checcucci, 2011 from the United Arab Emirates, and *K.agnellii* Taiti & Montesanto, 2018 from Djibouti. The new species differs from *K.rostratus* and *K.vanharteni* in having an interrupted frontal margin in the cephalothorax, and from *K.tamei*, *K.korshunovi*, and *K.agnellii* in having the inner lobe of schisma longer than outer one and the distal part of the male pleopod 1 endopodite bent inward with pointed apex. *Koweitoniscusshafieii* was found in southern Iran and according to current knowledge appears to be endemic to this region.

######## Distribution.

Iran (endemic): Jiroft district.

###### Genus *Somalodillo* Taiti & Ferrara, 1982

**Type species.***Somalodillosquamatus* Taiti & Ferrara, 1982 by original designation.

####### Somalodillo

Taxon classificationAnimaliaIsopodaEubelidae

sp.

Figure 6

######## Material examined.

Sistan va Balouchistan, [17] 1 ♀, Chabahar, 9 Feb. 2009, leg. E. Entezari (PCGMK 2111).

######## Remarks.

**Figure 6.**
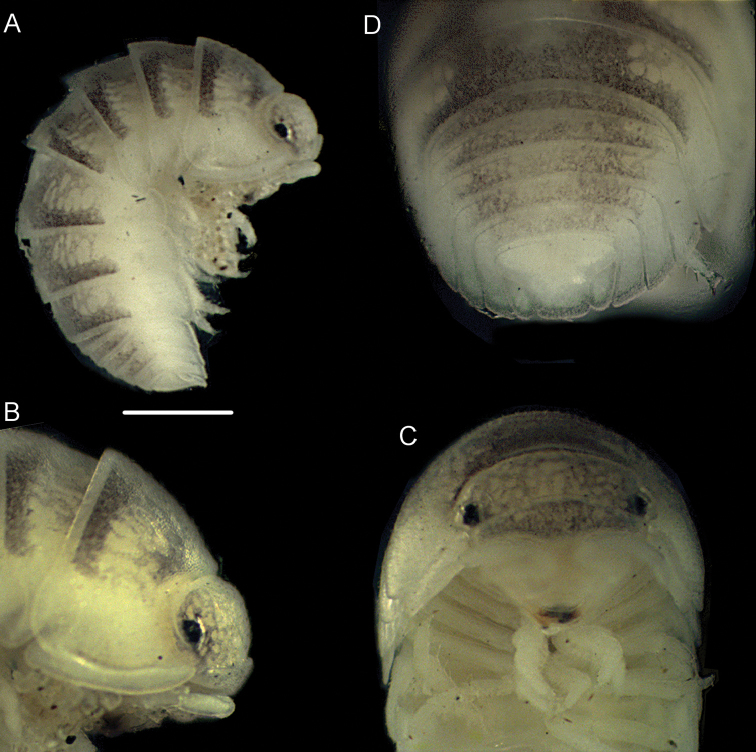
*Somalodillo* sp. **A** lateral view **B** cephalothorax and pereonite 1–2 in lateral view **C** cephalothorax in frontal view **D** pleon in dorsal view. Scale bar: 1 mm.

This is the first record of the genus *Somalodillo* reported from Iran. According to Taiti & Ferrara (1982; 2004), pereonite 1 with a schisma and sulcus arcuatus (Figure 6A–C), the telson with a rectangular distal part, uropod with minute exopodite inserted dorsally close to distal margin (Figure 6D), and pleopod exopodite 2 with monospiracular lungs clearly identify the specimen as a member of the genus *Somalodillo*. With no available male specimens, it was not possible to identify this female to specific level.

## Discussion

Despite several contributions on the terrestrial isopod fauna of Iran, especially in recent years, the knowledge on this taxon is relatively poor. In the present work, four eubelid species were found restricted to south and south-western Iran. The broad distribution of *Koweitoniscustamei* can be explained by the range expansion of the species to the south-western part of Iran while *Periscyphisvittatus* and *Somalodillo* sp. are most probably introduced to Iran by human activities. The new species, *Koweitoniscusshafieii*, is endemic to southern Iran. It seems that southern Iran represents the northernmost border for distribution of eubelid terrestrial isopods in the region. This work expands our knowledge on the oniscidean fauna of Iran, adding two genera and three species to the fauna of the country. Prior to the present study, 41 species were reported from Iran and this contribution raised the number to 44, which is still far from the real number of species probably present in the country.

## Supplementary Material

XML Treatment for Periscyphis vittatus

XML Treatment for Koweitoniscus tamei

XML Treatment for Koweitoniscus shafieii

XML Treatment for Somalodillo
